# Epidemiology, Characteristic, and Prognostic Factors of Primary Sporadic Intradural Malignant Peripheral Nerve Sheath Tumor in the Spinal Canal: A Systematic Literature Review

**DOI:** 10.3389/fonc.2022.911043

**Published:** 2022-07-08

**Authors:** Yue Cao, Yu-Bo Wang, Yang Bai, Xuan-yu Tan, Cheng-yuan Ma, Yong Chen, Hong-quan Yu, Hai-Yang Xu, Gang Zhao

**Affiliations:** Department of Neurosurgery, the First Hospital of Jilin University, Changchun, China

**Keywords:** malignant peripheral nerve sheath tumor, intradural, spinal, diagnosis, treatment, prognosis

## Abstract

**Purpose:**

Primary sporadic intradural malignant peripheral nerve sheath tumor (MPNST) in the spinal canal is a type of rare neoplasm with challenging diagnosis and therapy. The overall prognosis of this tumor is markedly different from that of the usual spinal intradural tumors. The purpose of this systematic review is to reduce the misdiagnosis and enhance the prognosis of the disease by reviewing the literature.

**Methods:**

PubMed, Medline, and Embase databases were searched for articles in English language published from 1980 to May 2021, yielding 500 potentially relevant articles. The keywords were as follows: “spinal”, “malignant peripheral nerve sheath tumor”, “neurosarcoma”, “malignant schwannoma”, and “malignant neurofibroma”. Thirteen papers met the eligibility criteria, including 55 cases with spinal intradural primary sporadic MPNSTs, which were confirmed by post-operation pathology. We further analyzed the clinical manifestations, radiological manifestations, pathological features, comprehensive treatment strategies, and prognosis.

**Results:**

Fifty-five spinal intradural primary sporadic MPNSTs from 30 (54.5%) male and 25 (45.5%) female patients with an average age at diagnosis of 40 years (range, 3–70 years) were included in the study. The most common clinical manifestations were local or radicular pain and motor disturbance. All tumors had significant enhancement and heterogeneous enhancement was more common. Out of 18 lesions, 14 were diagnosed as high grade and the remaining 4 were diagnosed as low grade. The ki-67 labeling index ranged from 5% to 60%. The median recurrence and survival time were 36 and 72 months, respectively. The log-rank tests indicated that significant predictors of OS were patient age (≤30 vs. >30 years) at the time of diagnosis and the presence of metastatic disease, and similar analyses for RFS demonstrated that the presence of metastatic disease was the only significant predictor (60 vs. 10 months). The multivariate Cox proportional hazards regression analysis revealed that absence of metastasis was an independent factor for predicting a favorable prognosis.

**Conclusions:**

Spinal intradural primary sporadic MPNSTs are challenging malignant tumors without a systematic treatment plan. The factors affecting its prognosis are not clear. Even after surgical treatment and adjuvant treatment, the recurrence rate and mortality rate are still high. Clinicians should be alert to the possibility of this disease and achieve early detection and treatment.

## Introduction

Malignant peripheral nerve sheath tumor (MPNST) is a highly malignant soft tissue tumor originated from mesenchymal cells and mainly distributed in the trunk, limbs, head and neck, and other areas of peripheral nerve distribution. MPNST (1 case in ten million) is an unusual disease and represents 2% to 4% of all soft tissue sarcomas and 23% to 51% of these tumors were associated with neurofibromatosis type 1 (NF1) (1). Spinal MPNSTs accounted for 2%–3% of all MPNSTs (2). Primary sporadic intradural MPNST in the spinal canal is even more exceptional, and it is easy to be misdiagnosed as central nervous system tumors or other types of soft tissue sarcomas. En bloc resection with a wide margin with adjuvant radiotherapy is considered as the first line for the therapy of non-spinal MPNSTs, and the implementation of this strategy is significant but not easy in the management of intradural MPNSTs. Research on the benefit of adjuvant chemotherapy is limited. In addition, compared to the usual spinal intradural tumors, overall prognosis of this tumor is distinctly different. We summarized 55 cases in the previous literature and analyzed their pathogenesis, clinical characteristics, imaging manifestations, differential diagnosis, surgical interventions, and pathological features to reduce the misdiagnosis and enhance the prognosis.

## Materials and Methods

### Literature Search

We searched the PubMed, Medline, and Embase databases for spinal MPNST-related articles. We have reviewed English literature in English language published from 1980 to May 2021. Search strategy was based on the following medical subject headings (MeSH) and keywords: “spinal”, “malignant peripheral nerve sheath tumor”, “neurosarcoma”, “malignant schwannoma”, and “malignant neurofibroma”. Inclusion criteria were as follows: (i) published in English, (ii) MPNST identified by pathological examination, (iii) some or all of the intradural tumors, and (iv) management options including subtotal resection, gross total resection, radiotherapy, chemotherapy, or combined treatments. We excluded the following three situations from our study: (i) malignant transformation in NF1, (ii) malignant transformation of other tumors like schwannoma or gangliocytoma, and (iii) radiotherapy-induced neoplastic lesions.

### Article Selection

**Figure 1:**
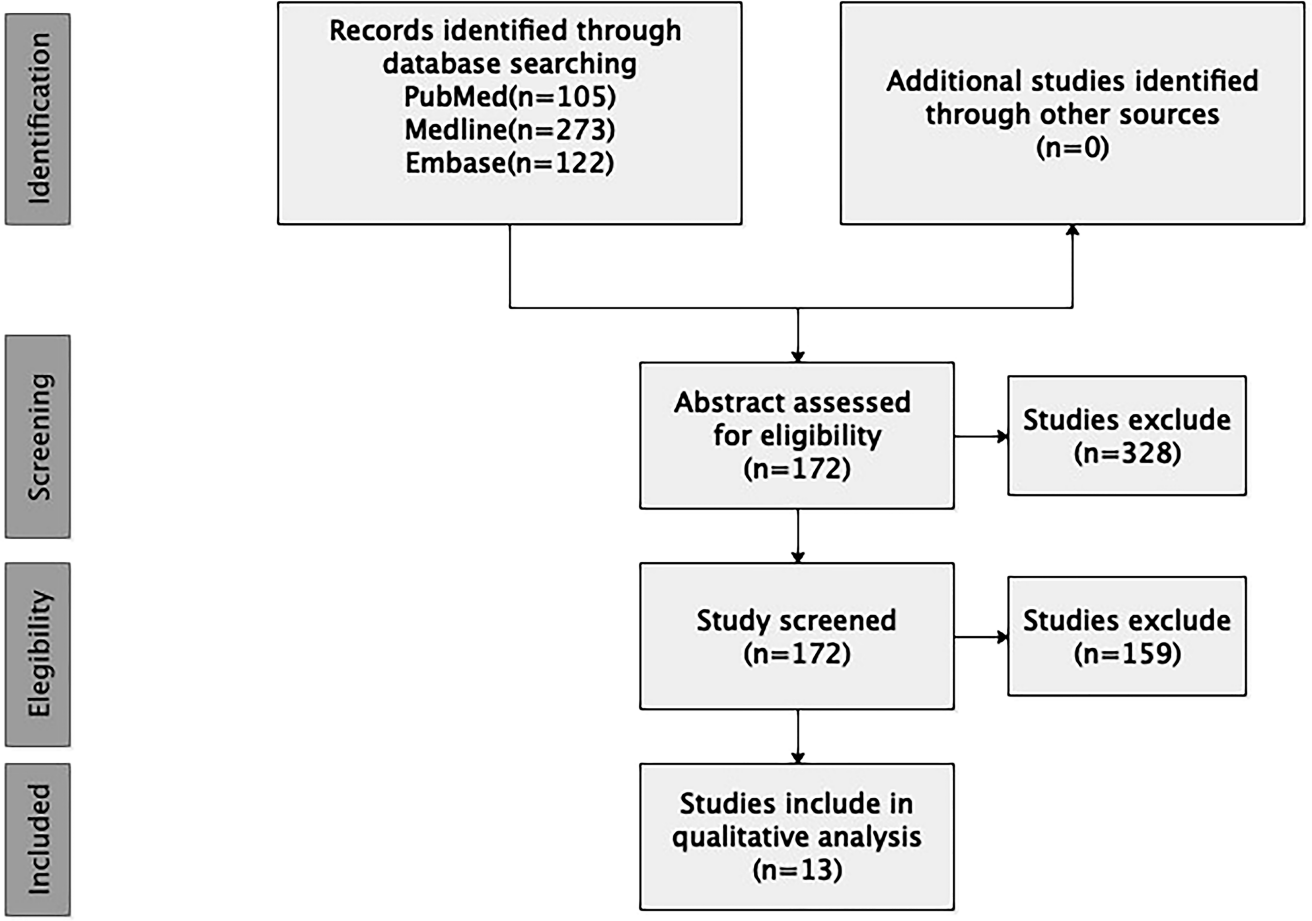
PRISMA flow diagram.

The search yielded 500 unique articles. Two authors reviewed each article title and abstract, and reached consensus regarding article eligibility based on the inclusion/exclusion criteria. A total of 13 papers including 55 cases with spinal intradural primary sporadic MPNSTs, which were confirmed by post-operation pathology, met all criteria and were included in the final review (**Figure 1**).

### Data Extraction and Analysis

We further analyzed the clinical manifestations, radiological manifestations, pathological features, comprehensive treatment strategies, and prognosis. Moreover, relapse-free survival (RFS) period was defined as the time from tumor resection to tumor relapse on imaging, and total survival period (OS) was defined as the time from tumor resection to death. RFS and OS curves were calculated by the Kaplan–Meier method. Log-rank test was adopted in the single-factor analysis to assess the intergroup differences. All variables with a significant result in the univariate Cox proportional hazard regression analysis were included in the following multivariate analysis. The hazard ratios (HR) and 95% confidence intervals (CIs) were estimated to identify the independent prognostic factors associated with RFS and OS in patients with primary sporadic intradural MPNST. A *p*-value of ≤0.05 was considered statistically significant.

## Results

### Clinical Data

Fifty-five spinal intradural primary sporadic MPNSTs [6 cervical (10.9%), 12 thoracic (21.8%), 6 lumbar (10.9%), 2 sacral (3.6%), and 29 unknown (52.7%)] from 30 (54.5%) male and 25 (45.5%) female patients with an average age at diagnosis of 40 years (range, 3–70 years) were included in the study. The maximum diameter of the tumors ranged from 1 cm to 9 cm. The most common clinical manifestations were local or radicular pain and motor disturbance. The mean duration of pre-operative clinical history was 12.6 months (range, 0.5–108 months) in 24 patients with relevant information. On T1-weighted imaging, 9 lesions appeared as isointense (9/16, 56.3%), and 7 lesions appeared as hypointense (7/16, 43.8%) signals. On T2-weighted imaging, 7 lesions were isointense (7/20, 35.0%), and 13 lesions were hypointense (13/20, 65.0%). Twenty-two cases recorded enhanced MRI information following gadolinium administration: The most common shape of tumors was oval (14/22, 63.6%), followed by irregular (4/22, 18.2%) and dumbbell (4/22, 18.2%); 15 tumors exhibited relatively clear boundaries (15/22, 68.2%), while 7 tumors exhibited obscure boundaries (7/22, 31.8%). All tumors had significant enhancement and heterogeneous enhancement was more common (11 vs. 3). Only 3/26 cases showed bone destruction on imaging. The demographic and clinical characteristics of these patients are summarized in **Table 1**.

**Table 1:** The demographic and clinical characteristics of these patients.

Study	Year	Nb		Location	Age (years)	Gender	Clinical symptoms	History (months)	Maximum diameter (cm)	Shape	Boundary of tumor	T1W1	T2W2	Enhancement	Bone destruction
Honda et al. (2)	2020	1	1	C5–C6	56	F	lt UE numbness and weakness	NA	NA	Dumbbell	Obscure	NA	Hyperintense	Yes	No
Chen et al. (3)	2019	8	1	T11/12	21	M	LE pain, low back pain	12	3	Oval (4/8), irregular (3/8), dumbbell (1/8)	Clear (5/8), obscure (3/8)	Isointense (4/8), hyperintense (4/8)	Isointense (3/8), hyperintense (5/8)	Heterogeneous enhancement (5/8), homogeneous enhancement (3/8)	Yes (1/8), no (7/8)
			2	L3–S1 (cauda equina)	29	F	rt LE numbness and weakness	6	4						
			3	L3–L4 (cauda equina)	52	M	low back pain	8	7.2						
			4	T2–L1	47	M	lt LE pain and weakness	1	4.6						
			5	C1–C3	39	F	lt UE and LE numbness and weakness	3	6.5						
			6	T6–T8	68	M	LE weakness	3	4.7						
			7	C5–C6	53	F	UE pain	6	3.2						
			8	T11	46	M	LE weakness	1	3						
Bettaswamy et al. (4)	2017	1	1	T8–T9	7	M	Low back pain	2	9	Dumbbell	Clear	Isointense	Isointense	Yes	Yes
Ghailane et al. (5)	2017	1	1	T12–L1	70	M	lt LE pain, low back pain	24	3.2	Dumbbell	Clear	Isointense	Isointense	Heterogeneous enhancement	No
Chou et al. (6) (multicenter study without individual information)	2017	29	29	NA	5–47 (mean 40)	M (17/29) F(12/29)	Pain (27/29), pathological fracture (2/29)	NA	NA						
Baharvahdat et al. (7)	2015	1	1	C1–T1	3	F	Back pain, UE and LE weakness	1	NA	Oval	Obscure	Isointense	Hyperintense	Heterogeneous enhancement	Yes
Thomas et al. (8)	2014	1	1*	Cauda equina	49	M	Low back pain, constipation, LE pain and weakness	0.5	NA	Oval	Obscure	Hyperintense	Isointense	Heterogeneous enhancement	No
Li et al. (9)	2014	1	1	T12–L1	33	F	Low back pain, rt LE pain	1	3.4	Oval	Clear	Isointense	Hyperintense	Heterogeneous enhancement	No
Yone et al. (10)	2004	1	1	L3–L5 (cauda equina)	4	M	lt LE pain, low back pain	NA	6	Oval	Clear	Isointense	Isointense	Heterogeneous enhancement	No
Celli et al. (11)	1995	5	1	T2	52	F	Pain, motor disturbance	8	1	Oval	Clear	NA		Yes	No
			2	L4 (cauda equina)	68	F	Pain, motor disturbance	9	2	Oval	Clear	NA		Yes	No
			3	L3 (cauda equina)	43	M	Pain	3	1	Oval	Clear	NA		Yes	No
			4	T11	36	F	Pain	5	3	Oval	Clear	NA		Yes	No
			5	T7	30	M	Pain, motor disturbance	72	3	Oval	Clear	NA		Yes	No
Seppälä et al. (12)	1993	3	1	Lumbar	13	M	Low back pain	6	NA						No
			2	Upper thoracic	23	F	Back pain	4	NA						No
			3	Lower cervical	37	F	Neck pain	12	NA						No
Valdueza et al. (13)	1991	2	1	T10–T12	43	F	Low back pain, LE weakness	1	NA	Irregular	Obscure	Hyperintense	Hyperintense	Yes	No
			2*	C4–C6	70	F	Neck pain, rt UE pain	6	NA	Oval	Clear	Hyperintense	Hyperintense	Heterogeneous enhancement	No
Thomeer et al. (14)	1981	1	1	Cauda equina	42	M	Low back pain, lt LE pain	108	NA						No

NA: not available; lt: left; rt: right; UE: upper extremity; LE: lower extremity; * two relapse-free survivors with a follow-up of less than 2 years.

### Pathological Features and Therapy

Immunohistochemical examinations revealed that S-100 protein was positive in 15/17 cases, vimentin in 10/14 cases, glial fibrillary acidic protein (GFAP) in 5/14 cases, desmin in 5/9 cases, epithelial membrane antigen (EMA) in 2/10 cases, cytokeratin in 1/8 cases, CD34 in 5/9 cases, and anti-smooth muscle antibody (SMA) in 5/9 cases. Based on the WHO classification, 14/18 lesions were diagnosed as high grade and the remaining 4 were diagnosed as low grade. The ki-67 labeling index ranged from 5% to 60%. All patients underwent microsurgical treatment. Eight patients received subtotal resection (8/27, 29.6%), and 19 patients received gross total resection (19/27, 70.4%). Thirty-three patients underwent postoperative radiotherapy and 14 patients underwent postoperative chemotherapy. The pathological features and therapy of these patients are summarized in **Table 2**.

**Table 2:** The pathological features and therapy of these patients.

Study	Year	Nb		Grade	Pathology									Surgery		Postoperative radiotherapy	Postoperative chemotherapy
					S-100	Vimentin	Desmin	GFAP	EMA	Cytokeratin	CD34	SMA	Ki-67				
Honda et al. (2)	2020	1	1	IV	NA									Dorsal standard midline approach	STR	Yes	No
Chen et al. (3)	2019	8	1	Low grade (3/8) high grade (5/8)	+ (6/8)	+ (5/8)	+ (4/8)	+ (3/8)	+ (2/8)	+ (1/8)	+ (6/8)	+ (4/8)	5%–60% (low 5-10%, mean 6.8%) (high 20%–60%, mean 40%)	Dorsal standard midline approach	GTR	No	Yes
			2												GTR	Yes	No
			3												GTR	Yes	No
			4												STR	Yes	Yes
			5												STR	Yes	No
			6												GTR	NA	NA
			7												STR	Yes	No
			8												GTR	NA	NA
Bettaswamy et al. (4)	2017	1	1	NA										Posterolateral thoracotomy approach	GTR	Yes	No
Ghailane et al. (5)	2017	1	1	IV	+	NA	+	NA						Dorsal standard midline approach	GTR	No	No
Chou et al. (6) (multicenter study without individual information)	2017	29	29	NA												Yes (19/29)	Yes (10/29)
Baharvahdat et al. (7)	2015	1	1	NA	+	+	NA	_	_	NA				Dorsal standard midline approach	STR	No	No
Thomas et al. (8)	2014	1	1*	NA	+	+	NA						7-10%	Dorsal standard midline approach	STR	No	No
Li et al. (9)	2014	1	1	NA	+	+	NA	_	_	NA	+	_	NA	Dorsal standard midline approach	STR	Yes	No
Yone et al. (10)	2004	1	1	NA	+	+	NA					+	NA	Dorsal standard midline approach	GTR	Yes	Yes
Celli et al. (11)	1995	5	1	IV	NA									NA	GTR	No	No
			2	IV	NA									NA	GTR	No	No
			3	IV	NA									NA	GTR	No	No
			4	IV	NA									NA	GTR	No	No
			5	IV	NA									NA	GTR	No	No
Seppälä et al. (12)	1993	3	1	NA	_	+	NA	_	NA					Dorsal standard midline approach	GTR	Yes	No
			2	NA	_	_	NA	+	NA					Dorsal standard midline approach	GTR	Yes	No
			3	NA										Dorsal standard midline approach	GTR	Yes	No
Valdueza et al. (13)	1991	2	1	III	+	NA		_	NA					Dorsal standard midline approach	STR	Yes	No
			2*	III	+	NA		+	NA					Dorsal standard midline approach	GTR	No	No
Thomeer et al. (14)	1981	1	1	II	NA									Dorsal standard midline approach	GTR	Yes	Yes

GTR, gross total resection; STR, subtotal resection.

### Follow-Up and Prognosis

The average follow-up period was 31.4 months, with a range of 0.3–120 months. During the follow-up period, 29 patients suffered from a local recurrence (29/55, 52.7%), and 11 patients experienced metastasis (11/26, 42.3%). The mean RFS was 30.8 months. Twenty-six patients died during the study period (26/55, 47.3%). Except for two relapse-free survivors with a follow-up of less than 2 years, 2-year recurrence rate and 2-year mortality rate were 43.4% (23/53) and 41.8% (22/53), respectively. The follow-up and prognosis of these patients are summarized in **Table 3**.

**Table 3:** The follow-up and prognosis of these patients.

Study	Year	Nb		Follow-up time (months)	Recurrence	Metastasis	Outcome
Honda et al. (2)	2020	1	1	36	Yes	No	Alive
Chen et al. (3)	2019	8	1	56	Yes	No	Died
			2	21	No	Lung	Died
			3	82	Yes	No	Died
			4	19	Yes	No	Died
			5	160	Yes (at 120 months)	No	Died
			6	15	Yes	No	Died
			7	10	Yes	Lung	Died
			8	28	No	No	Alive
Bettaswamy et al. (4)	2017	1	1	60	Yes	No	Alive
Ghailane et al. (5)	2017	1	1	10	Yes (at 3 months)	Yes	Died
Chou et al. (6) (multicenter study without individual information)	2017	29	29	24	Yes (11/29)	NA	Died (12/29)
Baharvahdat et al. (7)	2015	1	1	0.3	Yes	Brain, spinal	Died
Thomas et al. (8)	2014	1	1*	1.5	No	Brain, spinal	Alive
Li et al. (9)	2014	1	1	29	Yes (at 4 months)	Brain, spinal	Alive
Yone et al. (10)	2004	1	1	21	Yes (at 6 months)	Brain, spinal	Died
Celli et al. (11)	1995	5	1	72	No	No	Alive
			2	24	No	No	Alive
			3	72	No	No	Alive
			4	48	Yes	No	Alive
			5	14	No	Lung	Died
Seppälä et al. (12)	1993	3	1	7	Yes	Yes	Died
			2	8	Yes	Yes	Died
			3	72	Yes (at 24 months)	Yes	Died
Valdueza et al. (13)	1991	2	1	120	Yes (at 96 months)	No	Alive
			2*	7	No	No	Alive
Thomeer et al. (14)	1981	1	1	36	Yes	No	Alive

### Statistical Analysis

**Figure 2:**
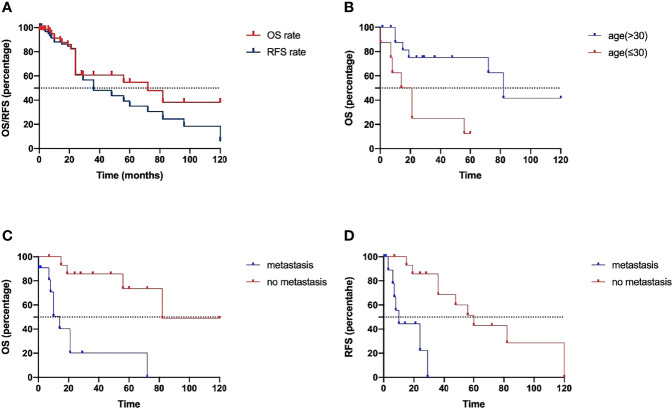
**(A)** The Kaplan–Meier curves of OS and RFS. The log-rank tests indicated that age at diagnosis **(B)** and presence or absence of metastasis **(C)** were the potential risk factors for OS, and presence or absence of metastasis **(D)** was also the potential risk factor for RFS.

The summary of patient data is shown in **Table 4**. The Kaplan–Meier curves of OS and RFS are shown in **Figure 2A**. The median recurrence and survival time were 36 and 72 months, respectively. The log-rank tests indicated that age at diagnosis (**Figure 2B**) and presence or absence of metastasis (**Figure 2C**) were the potential risk factors for OS, and presence or absence of metastasis (**Figure 2D**) was also the potential risk factor for RFS. The patients who were older than 30 years showed better OS, whose mean OS was 82 months, while the other patients had a mean OS of 17.5 months. The patients without metastasis had better OS and RFS, whose mean values were 82 months and 60 months, respectively. The mean OS and RFS of patients with metastasis were 14 months and 10 months. The patients without metastasis who were older than 30 years old have a better prognosis. The age at diagnosis and presence or absence of metastasis were included in the multivariate analysis. The multivariate Cox proportional hazards regression analysis revealed that absence of metastasis was an independent factor for predicting a favorable prognosis. The statistical results are summarized in **Table 5**.

**Table 4:** The summary of patient data.

Variables	Number	%
**Gender (*n* = 55)**		
Male	30	54.5%
Female	25	45.5%
**Age at diagnosis (years, *n* = 55)**		
Mean	40	
Range	3–70	
≤30	8	14.5
>30	18	32.7
Unknown	29	52.7%
**Location (*n* = 55)**		
Cervical	6	10.9
Thoracic	12	21.8
Lumber	6	10.9
Sacral	2	3.6
Unknown	29	52.7
**History (months, *n* = 24)**		
Mean	12.6	
Range	0.5–108	
≤6	16	66.7
>6	8	33.3
**Size (cm, *n* = 17)**		
Range	1–9	
≤3	10	58.8
>3	7	41.2
**Shape (*n* = 22)**		
Oval	14	63.6
Irregular	4	18.2
Dumbbell	4	18.2
**T1-weighted (*n* = 16)**		
Isointense	9	56.3
Hypointense	7	43.8
**T2-weighted (*n* = 20)**		
Isointense	7	35.0
Hypointense	13	65
**Boundary (*n* = 22)**		
Clear	15	68.2
Obscure	7	31.8
**Bone destruction (*n* = 26)**		
Yes	3	11.4
No	23	88.6
**Grade (*n* = 18)**		
Low grade	4	28.6
High grade	14	71.4
**S-100 (*n* = 17)**		
+	15	88.2
–	2	11.8
**Vimentin (*n* = 14)**		
+	10	71.4
–	4	28.6
**EMA (*n* = 10)**		
+	2	20.0
–	8	80.0
**CD34 (*n* = 9)**		
+	5	55.6
–	4	44.4
**SMA (*n* = 9)**		
+	5	55.6
–	4	44.4
**Desmin (*n* = 9)**		
+	5	55.6
–	4	44.4
**Cytokeratin (*n* = 8)**		
+	1	12,5
–	7	87.5
**Surgery (*n* = 27)**		
Subtotal resection	8	29.6
Gross total resection	19	70.4
**Postoperative adjuvant treatment (*n* = 53)**		
Radiotherapy	33	62.3
Chemotherapy	14	26.4
**Recurrence (*n* = 55)**		
Yes	29	52.7
No	26	47.3
**Metastasis (*n* = 26)**		
Yes	11	42.3
No	15	57.7
**Vital status (*n* = 55)**		
Alive	29	52.7
Died	26	47.3

**Table 5:** The results of the log-rank test, and univariate and multivariate Cox regression analysis.

Variable	Log-Rank Test		Univariate Analysis				Multivariate Analysis	
	OS	RFS	OS		RFS		OS	
	*p*-value	*p*-value	HR (95% CI)	*p*-value	HR (95% CI)	*p*-value	HR (95% CI)	*p*-value
Gender (*n* = 26)	0.279	0.356	Reference		Reference			
Male								
Female			0.545 (0.176–1.681)	0.291	0.588 (0.208–1.662)	0.317		
Age (>30) (*n* = 26)	0.004	0.221	Reference		Reference		Reference	
>30								
≤30			0.196 (0.057–0.670)	0.009	0.501 (0.169–1.539)	0.232	0.345 (0.095–1.256)	0.107
Location (cervical or not) (*n* = 26)	0.888	1	Reference		Reference			
Cervical								
Not cervical			1.097 (0.298–4.038)	0.889	1.003 (0.315–3.190)	0.996		
Boundary (*n* = 14)	0.894	0.685	Reference		Reference			
Obscure								
Clear			1.167 (0.120–11.341)	0.894	0.642 (0.074–5.583)	0.688		
Shape (oval or not) (*n* = 14)	0.762	0.633	Reference		Reference			
Oval								
Not oval			1.167 (0.120–11.341)	0.894	0.738 (0.155–3.508)	0.702		
Maximum diameter (>3 cm) (*n* = 17)	0.223	0.131	Reference		Reference			
>3 cm								
≤3 cm			2.567 (0.517–12.760)	0.249	3.162 (0.653–15.310)	0.152		
GTR vs. STR (*n* = 26)	0.538	0.652	Reference		Reference			
GTR								
STR			1.508 (0.400–5.692)	0.544	1.306 (0.405–4.213)	0.655		
Postoperative radiotherapy (*n* = 24)	0.953	0.276	Reference		Reference			
	
Yes								
No			0.964 (0.282–3.300)	0.954	2.013 (0.551–7.351)	0.29		
Postoperative chemotherapy (*n* = 24)	0.41	0.135	Reference		Reference			
	
Yes								
No			1.744 (0.448–6.788)	0.422	2.411 (0.723–8.038)	0.152		
Presence or absence of metastasis (*n* = 26)	<0.05	<0.05	Reference		Reference		Reference	
Metastasis								
Not metastasis			8.554 (2.254–32.464)	0.002	12.782 (2.529–64.605)	0.002	6.504 (1.579–26.796)	0.010

## Discussion

MPNSTs are highly aggressive and locally invasive rare malignancies with an incidence of 0.0001% in the general population and 3%–5% in patients with neurofibromatosis type 1 (NF1) (2). Lesions are most frequently found on the trunk, extremities, and head and neck. There are three main forms of histogenesis of MPNSTs (15): half of the cases are sporadic and derive from peripheral nerves that originate from Schwann cells or pluripotent cells of neural crest origin (sporadic type) (16); about 50%–60% MPNSTs occur in the malignant transformation of NF1 (NF1 type); and a few cases are radiotherapy-induced or malignant change of schwannoma and ganglioma. Thus, primary sporadic MPNST with an intradural occurrence of the spine outside the setting of neurofibromatosis was extremely rare and associated with an extremely rare diagnosis and an extremely poor prognosis in comparison to non-spinal MPNST. In our present research, we conducted a retrospective study to thoroughly analyze the pathogenesis, clinical characteristics, imaging manifestations, differential diagnosis, surgical interventions, pathological features, and prognosis of primary sporadic intradural MPNSTs.

We found only 55 cases of primary sporadic intradural MPNSTs without neurofibromatosis in our search to this date—more men than women (54.5% > 45.5%). The median age at diagnosis was 40 years, with a range of 3–70 years. As reported in the previous study, this kind of tumor occurred primarily in adults, which was largely consistent with those of our research. The disease history in our study had a median of 12.6 months, which was much longer than that found in previous reports (3). The thoracic spine was the most frequently affected area. Local or radicular pain and motor disturbance were the most common clinical symptoms, which were nonspecific and made a challenging diagnosis. Furthermore, MPNST can masquerade as common benign nerve sheath tumors on imaging (16, 17), which generally exhibit an isointense signal in T1-weighted imaging and a hyperintense signal in T2-weighted imaging. In the present investigation, there were still 43.8% of the tumors that showed hyperintensity in T1-weighted imaging. All tumors showed varying degrees of enhancement. Furthermore, MPNST did not show typical invasive growth (irregularly or obscure bordered) and destruction of surrounding osseous structures on the radiograph. Since MPNSTs show higher metabolic activity, 18F-FDG PET/CT may be helpful for the diagnosis (18). A tumor SUV is higher than that of normal liver tissue, which is considered to be a sensitive and specific index of MPNST (19). According to the authors’ experience, when the imaging findings are benign intraspinal tumors, but the adhesion between the tumor and the nerve is serious intraoperatively, the possibility of MPNST should be considered. Thus, we advocate that regardless of the clinical manifestation or imaging characteristics, surgeons should retain a high index of suspicion for an MPSNT, especially when excision is laborious during surgery. Spine MRI is essential in postoperative follow-up because of the high incidence of drop metastasis (20).

Surgical biopsy result is the gold standard and past medical history is an important diagnostic evidence. Pathological characteristics of spinal MPNST are high cellularity with spindle-shaped cells, nuclear atypia, necrosis, endothelial proliferation, and so on (7). HE staining was characterized by “marble-like” spindle-shaped tumor cells, alternating between dense and loose areas, and arranged in bundles or swirls (21). There were no ganglion cells in the tumor. S-100 is a characteristic protein of primary MPNST, but when the tumor is recurrent or highly malignant, the positive rate of S-100 is significantly decreased (10, 22). S-100 was negative only in 2 patients in our study; hence, the clinical significance of it needs to be further investigated. Positive CD34 indicates the presence of heterogeneous cellular components in the tumor. In addition, high-grade MPNST often expresses p53. Loss of SMARCB1 expression plays an important role in the occurrence and development of MPNST (21). Due to incomplete sample information, we only made a summary of the pathological results. Except for the surgical biopsy result, an accurate diagnosis of primary spinal intradural MPNSTs depends on the exclusion of metastasis, malignant transformation, radiotherapy-induced tumor, and NF1. Further study of molecular pathology is an effective way for diagnosis and treatment. In addition, the analysis of cancer stem cells and genetics in MPNSTs is helpful to design new treatment schemes (23). Spyra et al. suggested the increased expression of CD133, Oct4, and Nestin, and decreased markers of NCAM and CD90 (24). Genetic mutations such as SUZ12, EED, BRAF^V600E^, and TP53 have been reported in sporadic MPNSTs (25–28).

Due to the lack of a large amount of clinical data about primary sporadic intradural MPNSTs, there is no mature and effective treatment plan at present. A reasonable stage and risk grouping of MPNSTs is beneficial to the subsequent management (18). Surgical resection is the mainstay of treatment currently, while the outcomes of surgical management are widely disparate (8). Generally speaking, there are two types of resections: one is piecemeal resection, which means that an intralesional resection involved violation of the tumor capsule, and the other is en bloc resection, which refers to the circumferential separation of the tumor without violation of its border or capsule, and can be categorized into wide margin and marginal margin according to the different surgical margin (29). Radical en bloc resection with wide margins is a difficult but significant factor in tumor control and future prognosis (30). The surrounding vital structures, including critical nerves and blood vessels, restrict the extent of the resection range. Chou et al. classified the surgical technique for spinal MPNSTs as Enneking appropriate (EA) or Enneking inappropriate (EI) to investigate the effects of two types on recurrence and survival (6). EA surgery is en bloc resection with wide or marginal margins and EI surgery is a piecemeal or an intralesional resection. In their study, there was no difference in recurrence or survival rate based on the two resection techniques. They also suggested that EA resection was not necessary to improve the overall survival because of the spread along nerves and multiple skip metastases, but better progression-free period may be obtained. However, the benefit of EA resection may be undermined by operation-related structure damage compared to EI resection (especially intralesional piecemeal resections). Another study suggested that the reason of relapse and metastasis in piecemeal total resection probably originated from tumor cell contamination in the surgical field (3). In our research, the present results suggest that the extent of surgical resection may not affect overall or local relapse-free survival. Although piecemeal total resection may not yield a conclusive tumor-free margin, it may alleviate symptoms, achieve sufficient volume reduction and bring greater benefit to patients. A reasonable surgical design is an effective and primary way to gain time for subsequent treatment. The best adjuvant treatment remains poorly defined due to the lack of prospective trials. Previous literature suggests that adjuvant radiotherapy after surgery could be an effective treatment for patients, especially in lesions larger than 5 cm in size or with residual tumor, which is critical in the prognosis of primary spinal intradural MPNSTs (2, 7, 8, 31). However, our study revealed that radiotherapy is ineffective in controlling recurrence and does not appear to affect overall survival, which may be due to the bias caused by the fact that more aggressive tumors are more likely to undergo radiotherapy. Additionally, radiotherapy had the risk of increasing the mutational burden of the tumor (23). Further exploration is required to elucidate the effect of surgical type and adjuvant radiotherapy. At present, there is no consensus on chemotherapy and it requires personalized design for MPNSTs. Chemotherapy did not show benefit in our present study. In view of the resistance of MPNSTs to traditional chemotherapy (32), targeted therapy is a new therapeutic strategy and direction (33). Some other new treatments, like carbon ion radiotherapy (CIRT), are currently under study and being explored (2).

The clinical outcome of primary sporadic intradural MPNSTs is poor (34). The rate of metastasis at the time of initial diagnosis is 10.4% (35), and 5-year survival rate is 42%–50% in sporadic cases (36). In our research, the rate of tumor recurrence was 52.7%, and the rate of tumor metastasis was 42.3%. The 2-year recurrence rate and the 2-year mortality rate were 43.4% and 41.8%, respectively. The median recurrence and survival time were 36 and 72 months, respectively. In this retrospective study, we found age and presence of metastasis as two prognostic factors, which could influence the OS and RFS. The patients who were older than 30 years showed better OS than the other patients. The patients without metastasis had better OS and RFS. Furthermore, the multivariate Cox proportional hazards regression analysis revealed that absence of metastasis was an independent factor for predicting a favorable prognosis. However, the patients’ gender, the position of the tumor, surgery, adjuvant therapy, and many other factors did not appear to affect the prognosis.

## Limitation

The study is limited by its small sample size, and some data are not detailed and complete. The criterion of “exclusion of tumors that had undergone secondary transformation” is perhaps misleading. It is possible that some patients may have had undiagnosed schwannomas/other tumors that underwent secondary transformation and were only diagnosed at that point. More relevant clinical data need to be screened, collected, and studied.

## Conclusion

Primary sporadic intradural MPNSTs are aggressive malignant tumors with high mortality and morbidity rates, even after formal treatment. It is difficult to make a diagnosis based on clinical and imaging findings alone. Surgical resection and pathological examination are necessary. The benefit of radiotherapy and chemotherapy treatments remains controversial. In our present study, early detection of diseases in adults may predict better clinical outcomes. However, we should be aware that further studies with larger cohorts are needed to explore the prognostic factors and reasonable treatment plans.

## Data Availability Statement

The original contributions presented in the study are included in the article/supplementary material. Further inquiries can be directed to the corresponding authors.

## Author Contributions

YC and Y-BW conducted the data analysis, interpreted the data, and wrote the main manuscript. YB, C-YM, and H-QY supervised the data analysis and interpreted the data. H-YX and GZ designed the research and critically revised the article. All other co-authors helped to interpret the data and critically reviewed the article. All authors approved the final article for submission.

## Funding

This work was supported by National Nature and Science Foundation of China (81772684), the S&T Development Planning Program of Jilin Province (20200201469JC, 20200201613JC, and 20200201388JC), Foundation from the Development and Reform Commission of Jilin Province (Grant No. 2017C059-2), and Chinese People’s Brain Neural Network Research and Innovation Cooperation Platform Construction Project.

## Conflict of Interest

The authors declare that the research was conducted in the absence of any commercial or financial relationships that could be construed as a potential conflict of interest.

## Publisher’s Note

All claims expressed in this article are solely those of the authors and do not necessarily represent those of their affiliated organizations, or those of the publisher, the editors and the reviewers. Any product that may be evaluated in this article, or claim that may be made by its manufacturer, is not guaranteed or endorsed by the publisher.

## References

[1] Martin E Muskens IS Coert JH Smith TR Broekman MLD . Treatment and Survival Differences Across Tumor Sites in Malignant Peripheral Nerve Sheath Tumors: A SEER Database Analysis and Review of the Literature. Neuro-Oncol Pract (2019) 6:134–43. doi: 10.1093/nop/npy025 PMC6656331 31386019

[2] Honda A Iizuka Y Okamoto M Shiba S Koshi H Mieda T . Malignant Peripheral Nerve Sheath Tumor of the Cervical Spine Treated With Surgical Resection Followed by X-Ray Radiotherapy or Carbon Ion Radiotherapy: A Report of Three Cases. Spine Surg Relat Res (2020) 4:269–73. doi: 10.22603/SSRR.2019-0100 PMC7447340 32864495

[3] Chen J Zheng Y Chen Z Fan F Wang Y . Clinical Presentation and Long-Term Outcome of Primary Spinal Intradural Malignant Peripheral Nerve Sheath Tumors. Clin Neurol Neurosurg (2019) 185:105484. doi: 10.1016/j.clineuro.2019.105484 31421588

[4] Bettaswamy G Ambesh P Kumar R Sahu RN Das KK . Multicompartmental Primary Spinal Extramedullary Tumors : Value of an Interdisciplinary Approach. Asian J Neurosurg (2017) 12:674–80. doi: 10.4103/ajns.AJNS PMC5652094 29114282

[5] Ghailane S Fauquier S Lepreux S Le Huec JC . Malignant Triton Tumor: Grand Round Presentation of a Rare Aggressive Case Thoracolumbar Spine Tumor. Eur Spine J (2019) 28:1448–52. doi: 10.1007/s00586-017-5277-2 28924675

[6] Chou D Bilsky MH Luzzati A Fisher CG Gokaslan ZL Rhines LD . Malignant Peripheral Nerve Sheath Tumors of the Spine: Results of Surgical Management From a Multicenter Study. J Neurosurg Spine (2017) 26:291–8. doi: 10.3171/2016.8.SPINE151548 27834629

[7] Baharvahdat H Ganjeifar B Roshan NM Baradaran A . Spinal Intradural Primary Malignant Peripheral Nerve Sheath Tumor With Leptomeningeal Seeding: Case Report and Literature Review. Turk Neurosurg (2018) 28:317–22. doi: 10.5137/1019-5149.JTN.16782-15.1 27593814

[8] Thomas JG Lincoln C Goodman JC Gopinath SP . Malignant Peripheral Nerve Sheath Tumor of the Cauda Equina With Craniospinal Metastasis. J Clin Neurosci (2014) 21:2239–42. doi: 10.1016/j.jocn.2014.02.028 24986157

[9] Li Y Fan F Xu J An J Zhang W . Primary Malignant Peripheral Nerve Sheath Tumor of the Spine With Acute Hydrocephalus: A Rare Clinical Entity. J Neurosurg Spine (2014) 21:367–71. doi: 10.3171/2014.4.SPINE13739 24926928

[10] Yone K Ijiri K Hayashi K Yokouchi M Takenouchi T Manago K . Case Report Primary Malignant Peripheral Nerve Sheath Tumor of the Cauda Equina in a Child Case Report. Spinal Cord (2004) 42:199–203. doi: 10.1038/sj.sc.3101567 15001982

[11] Celli P Cervoni L Tarantino R Fortuna A . Primary Spinal Malignant Schwannomas: Clinical and Prognostic Remarks. Acta Neurochir (Wien) (1995) 135:52–5. doi: 10.1007/BF02307414 8748792

[12] Seppala MT Haltia MJJ . Spinal Malignant Nerve-Sheath Tumor or Cellular Schwannoma? A Striking Difference in Prognosis. J Neurosurg (1993) 79:528–32. doi: 10.3171/jns.1993.79.4.0528 8410221

[13] Valdueza JM Hagel C Westphal M Hänsel M Herrmann HD . Primary Spinal Malignant Schwannoma: Clinical, Histological and Cytogenetic Findings. Neurosurg Rev (1991) 14:283–91. doi: 10.1007/BF00383263 1791943

[14] Thomeer RT Bots GT van Dulken H Luyendijk W Helle P . Neurofibrosarcoma of the Cauda Equina. Case Rep J Neurosurg (1981) 54:409–11. doi: 10.3171/jns.1981.54.3.0409 7463146

[15] Lafemina J Qin LX Moraco NH Antonescu CR Fields RC Crago AM . Oncologic Outcomes of Sporadic, Neurofibromatosis-Associated, and Radiation-Induced Malignant Peripheral Nerve Sheath Tumors. Ann Surg Oncol (2013) 20:66–72. doi: 10.1245/s10434-012-2573-2 22878618 PMC3567771

[16] Wu OC Shammassian BH Chugh AJS Harbhajanka A Kasliwal MK . Ominous Occurrence of Spinal Intradural Primary Malignant Peripheral Nerve Sheath Tumor Four Decades Following Radiation Therapy for Testicular Seminoma. Case Rep Neurol Med (2020) 2020:1–8. doi: 10.1155/2020/1792582 PMC7007932 32047679

[17] Koeller KK Shih RY . Intradural Extramedullary Spinal Neoplasms: Radiologic-Pathologic Correlation. Radiographics (2019) 39:468–90. doi: 10.1148/rg.2019180200 30844353

[18] Knight SWE Knight TE Santiago T Murphy AJ Abdelhafeez AH . Malignant Peripheral Nerve Sheath Tumors—A Comprehensive Review of Pathophysiology, Diagnosis, and Multidisciplinary Management. Children (2022) 9:38. doi: 10.3390/children9010038 35053663 PMC8774267

[19] Assadi M Velez E Najafi MH Matcuk G Gholamrezanezhad A . PET Imaging of Peripheral Nerve Tumors. PET Clin (2019) 14:81–9. doi: 10.1016/j.cpet.2018.08.013 30420224

[20] Ziadi A Saliba I . Malignant Peripheral Nerve Sheath Tumor of Intracranial Nerve: A Case Series Review. Auris Nasus Larynx (2010) 37:539–45. doi: 10.1016/j.anl.2010.02.009 20399579

[21] Nakayama Y Watanabe M Suzuki K Usuda H Emura I Ogura R . Malignant Peripheral Nerve Sheath Tumor of the Trigeminal Nerve: Clinicopathologic Features in a Young Adult Patient. Neuropathology (2013) 33:541–6. doi: 10.1111/neup.12004 23279368

[22] Winslow N Abode-Iyamah K Kirby P Smith M Reddy C . Malignant Peripheral Nerve Sheath Tumor Arising in the Setting of Cervical Nerve Root Schwannomas. J Clin Neurosci (2015) 22:1696–9. doi: 10.1016/j.jocn.2015.05.016 26117359

[23] Somatilaka BN Sadek A McKay RM Le LQ . Malignant Peripheral Nerve Sheath Tumor: Models, Biology, and Translation. Oncogene (2022) 41:2405–21. doi: 10.1038/s41388-022-02290-1 PMC9035132 35393544

[24] Spyra M Kluwe L Hagel C Nguyen R Panse J Kurtz A . Cancer Stem Cell-Like Cells Derived From Malignant Peripheral Nerve Sheath Tumors. PLoS One (2011) 6:e21099. doi: 10.1371/journal.pone.0021099 21695156 PMC3113907

[25] Lee W Teckie S Wiesner T Ran L Prieto Granada CN Lin M . PRC2 is Recurrently Inactivated Through EED or SUZ12 Loss in Malignant Peripheral Nerve Sheath Tumors. Nat Genet (2014) 46:1227–32. doi: 10.1038/ng.3095 PMC4249650 25240281

[26] Ming Z Yuxuan W Sian J Sausen M McMahon K Sharma R . Somatic Mutations of SUZ12 in Malignant Peripheral Nerve Sheath Tumors. Nat Genet (2014) 46:1170–2. doi: 10.1038/ng.3116.Somatic PMC4383254 25305755

[27] Hirbe AC Pekmezci M Dahiya S Apicelli AJ Van Tine BA Perry A . BRAFV600E Mutation in Sporadic and Neurofibromatosis Type 1-Related Malignant Peripheral Nerve Sheath Tumors. Neuro Oncol (2014) 16:466–7. doi: 10.1093/neuonc/not248 PMC3922521 24366910

[28] Legius E Dierick H Wu R Hall BK Marynen P Cassiman J-J . TP53 Mutations are Frequent in Malignant NFI Tumors. Genes Chromosom Cancer (1994) 10:250–5. doi: 10.1002/gcc.2870100405 7522538

[29] Matsumoto Y Kawaguchi K Fukushi J Endo M Setsu N Iida K . Clinical Outcome and Prognostic Factors of Malignant Spinal Dumbbell Tumors. Spine Surg Relat Res (2018) 2:317–23. doi: 10.22603/ssrr.2018-0004 PMC6690099 31435541

[30] Vauthey JN Woodruff JM Brennan MF . Extremity Malignant Peripheral Nerve Sheath Tumors (Neurogenic Sarcomas): A 10-Year Experience. Ann Surg Oncol (1995) 2:126–31. doi: 10.1007/BF02303627 7728565

[31] Newell C Chalil A Langdon KD Karapetyan V Hebb MO Siddiqi F . Cranial Nerve and Intramedullary Spinal Malignant Peripheral Nerve Sheath Tumor Associated With Neurofibromatosis-1. Surg Neurol Int (2021) 12:1–5. doi: 10.25259/SNI_595_2021 35350820 PMC8942193

[32] Pervaiz N Colterjohn N Farrokhyar F Tozer R Figueredo A Ghert M . A Systematic Meta-Analysis of Randomized Controlled Trials of Adjuvant Chemotherapy for Localized Resectable Soft-Tissue Sarcoma. Cancer (2008) 113:573–81. doi: 10.1002/cncr.23592 18521899

[33] Nagabushan S Lau LMS Barahona P Wong M Sherstyuk A Marshall GM . Efficacy of MEK Inhibition in a Recurrent Malignant Peripheral Nerve Sheath Tumor. NPJ Precis Oncol (2021) 5:1–6. doi: 10.1038/s41698-021-00145-8 33580196 PMC7881142

[34] Wang T Yin H Han S Yang X Wang J Huang Q . Malignant Peripheral Nerve Sheath Tumor (MPNST) in the Spine: A Retrospective Analysis of Clinical and Molecular Prognostic Factors. J Neurooncol (2015) 122:349–55. doi: 10.1007/s11060-015-1721-5 25598015

[35] Miao R Wang H Jacobson A Lietz AP Choy E Raskin KA . Radiation-Induced and Neurofibromatosis-Associated Malignant Peripheral Nerve Sheath Tumors (MPNST)have Worse Outcomes Than Sporadic MPNST. Radiother Oncol (2019) 137:61–70. doi: 10.1016/j.radonc.2019.03.015 31078939

[36] Evans DGR Baser ME McGaughran J Sharif S Howard E Moran A . Malignant Peripheral Nerve Sheath Tumours in Neurofibromatosis. J Med Genet (2002) 39:311–4. doi: 10.1136/jmg.39.5.311 PMC1735122 12011145

